# Porphyrin as a Cryoprotectant for Graphene Oxide-Coated Gold Nanorods to Produce Conjugated Product with Improved Stability and Opto-Phototherapeutic Properties

**DOI:** 10.3390/pharmaceutics15112538

**Published:** 2023-10-27

**Authors:** Thabang Calvin Lebepe, Rodney Maluleke, Nande Mgedle, Oluwatobi Samuel Oluwafemi

**Affiliations:** 1 Centre for Nanomaterials Science Research, University of Johannesburg, Johannesburg 2028, South Africa; calvyn.tl@gmail.com (T.C.L.); rodney.maluleke@gmail.com (R.M.); nandemgedle@gmail.com (N.M.); 2 Department of Chemical Sciences, University of Johannesburg, Johannesburg 2028, South Africa

**Keywords:** freeze-drying, gold nanorods, graphene oxide, porphyrin

## Abstract

Graphene oxide (GO) as a coating material for gold nanorods (AuNRs) has gained interest in reducing toxicity and improving the photothermal profiling of AuNRs. However, there is still a challenge regarding the storage of colloidal suspensions of GO-coated AuNRs (GO@AuNRs). Hence, the conjugation of GO@AuNRs to meso-tetra-(4-sulfonatophenyl)porphyrin (TPPS_4_), an anionic water-soluble porphyrin, has been reported to enhance their re-dispensability and improve their phototherapeutic properties. The AuNRs and GO were synthesised using seed-mediated and Hummers’ methods, respectively. The GO@AuNRs were conjugated to TPPS_4_ and characterised using ultraviolet–visible–near-infrared (UV-Vis-NIR) spectroscopy, zeta analyser, dynamic light scattering (DLS), photoluminescence spectroscopy (PL), x-ray diffraction (XRD), high-resolution transmission electron microscopy (HRTEM) and Fourier-transform infrared spectroscopy (FTIR) before freeze-drying. The results showed that the AuNRs were sandwiched between GO and TPPS_4_. After freeze-drying, the freeze-dried conjugate was dispensed in deionised water without adding cryoprotectants and its properties were compared to those of the unfreeze-dried conjugate. The results showed that the freeze-dried conjugate contained similar optical properties to the unfreeze-dried conjugate. However, the bare GO@AuNRs showed a change in the optical properties after freeze-drying. These results revealed that porphyrin is an excellent additive to reduce the freeze-drying stress tolerance of GO@AuNRs. The freeze-dried conjugate also showed both singlet oxygen and photothermal properties of GO@AuNRs and porphyrin. These results indicated that the freeze-dried conjugate is a promising dual photodynamic and photothermal agent, and porphyrin can act as a cryoprotectant.

## 1. Introduction

Numerous attempts have been made to improve the application of AuNRs by applying different functionalisation strategies such as ligand exchange and single or multi-layer encapsulation approaches using polymers, graphene-based materials, proteins, and metal oxides [1,2,3,4]. However, some coatings limit the biomedical application of AuNRs because they lead to thermal instability, lack of versatility, and more toxicity risk of AuNRs. Current research has shown that conjugating, coating, or functionalising AuNRs with graphene oxide (GO) increases the surface area of the AuNRs. This modification advances the application of AuNRs as drug carriers [5,6] and as biocompatible photothermal stable material [2,5,7,8]. Furthermore, it has been shown that the cytotoxicity of AuNRs can be lowered by removing CTAB and encapsulating them with GO [8].

On the other hand, AuNRs have been functionalised with photosensitisers for different applications such as synergistic phototherapy [9,10,11,12,13] and as a sensor [14,15]. However, nanoparticle colloidal preservation in suspensions is still a major challenge. Many polymers have been used to improve their colloidal stability [16,17,18,19]. Freeze-drying, also known as lyophilisation or cryodesiccation technique, is also used to maintain the nanomaterials’ colloidal stability by converting them into a solid mass. This technique has been used widely to preserve constantly changing biological systems such as proteins, antibodies, plasma constituents, and others in the pharmaceutical, food, and agricultural industries for safe shipping and storage [20]. During lyophilisation, cryoprotectants such as mono- and disaccharides, surfactants, amino acids, and polymers are usually used to reduce stresses [20,21,22,23].

**Scheme 1:**
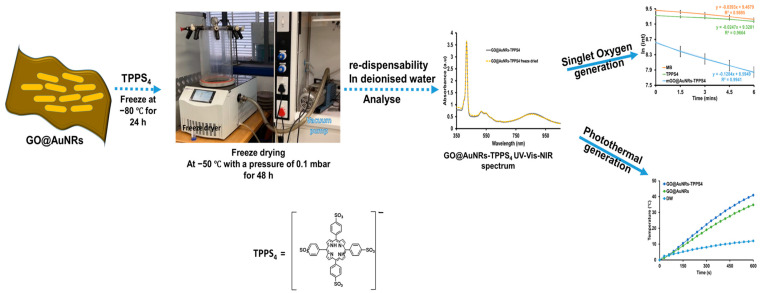
Schematic diagram of water-soluble porphyrin to enhance photo properties of GO-coated AuNRs as well as re-dispensability after freeze-drying.

Nonetheless, when GO is used to coat the AuNRs, the samples cannot be freeze-dried. This is because during the lyophilisation, the oxygen-containing functional groups are removed from GO, exposing AuNRs to lower temperatures and thus changing their shape. In addition, the sample cannot be redispersed in water because the oxygen-containing functional groups on the graphene are the reason for the easy dispensability of GO in water [24]. In this study, the GO-coated AuNRs were conjugated to meso-tetra-(4-sulfonatophenyl)porphyrin (TPPS_4_), a water-soluble porphyrin to enhance their re-dispensability. Water-soluble porphyrins like TPPS_4_ have been used to improve the solubility of nanomaterials [25,26], and because they are used for photodynamic applications, they can enhance the nanomaterials’ therapeutic applications for cancer and antimicrobial applications [27]. However, to the best of our knowledge, the effect of conjugating porphyrin to improve the GO-coated AuNRs (GO@AuNRs) as cryoprotectants has not been investigated. Unlike when polymers are used as cryoprotectants, conjugation with porphyrin will reduce stresses during lyophilisation and improve the therapeutic effect of GO@AuNRs. The as-synthesised conjugate (GO@AuNRs-TPPS_4_) was found to be easily re-dispersible in water after freeze-drying compared to GO@AuNRs. The conjugate photo-decay time was found to be quicker compared to TPPS_4_, even though the quantum yield was lower in the conjugate because of the GO@AuNRs. The singlet oxygen generation kinetics were found to be slower, and the conjugation did not affect the photothermal properties of GO@AuNRs. These results show that porphyrin can be used as a cryoprotectant, and the conjugate can be used as a dual phototherapeutic agent (Scheme 1).

## 2. Materials and Methods

### 2.1. Materials

Hydrogen tetra-chloroauric hydrate (HAuCl_4_.xH_2_O, 99.9%), sodium borohydride (NaBH_4_, 99%), silver nitrate (AgNO_3_, 99%), cetyltrimethylammonium bromide (CTAB, ≥99%), hydroquinone (HQ, %), sodium oleate (NaOL, ≥99%), hydrochloric acid (HCl, 12.1 M), graphite powder, sulfuric acid (H_2_SO_4_), sodium nitrate (NaNO_3_), potassium permanganate (KMnO_4_), peroxide (H_2_O_2_), sodium hydroxide (NaOH), polyvinylpyrrolidone (PVP), and methanol were purchased from Sigma-Aldrich, Kempton Park, South Africa. All solutions of gold salt, AgNO_3_, and NaBH_4_ were freshly prepared. All glasswares used in the experiments were cleaned and washed thoroughly with MilliQ water (15.0 MΩ cm @ 25 °C) and dried before use.

### 2.2. Characterisation Techniques

UV-Vis-NIR JASCO V-770 spectrophotometer (JASCO Corp., Tokyo, Japan) was used to measure the UV-Vis-NIR absorption spectra of the samples. The surface chemistry was investigated using Spectrum Two UATR spectrometer, Perkin Elmer(Beaconsfield, UK). The morphologies of the samples were captured by high-resolution transmission electron microscopy (HRTEM, JEOL 2010,200 KV, Tokyo, Japan). The sizes of the AuNRs and composites were measured from TEM images using ImageJ software (version 1.8.0). Zeta sizer (Microtrac Nanotrac Wave II, Microtrac MRB, Duesseldorf, Germany) was used to measure the surface charge of the materials. The fluorescence spectra of the samples were measured using Shimadzu spectrophotometer (RF-6000, Kyoto, Japan). For NIR irradiation, a dst11-LUMICS-808 nm 27W continuous Nd: YV04 air-cooled laser system (OsTech e. K., Berlin, Germany) with an optical fibre to deliver an 8 mm beam diameter was used.

### 2.3. Synthesis of Graphene Oxide

GO was prepared using a modified Hummers’ method [28]. Briefly, ice-cold concentrated H_2_SO_4_ (69 mL) was added to a mixture of graphite flakes (3.0031 g) and NaNO_3_ (1.5062 g) under continuous stirring in an ice bath. This was followed by the slow addition of KMnO_4_ (~10 g) and warmed to 35 °C while stirring for 30 min. Next, 138 mL of distilled water was added in portions, the temperature was increased to 95 °C, and it was stirred for 30 min. The solution was then cooled to room temperature, and the reaction was terminated by adding H_2_O_2_ (30 mL). The solution was left to settle, and the suspension was discarded, followed by pH neutralisation with distilled water and allowed to dry at room temperature. The obtained graphite oxide powder was exfoliated into GO sheets using ultrasonication.

### 2.4. Synthesis of Gold Nanorods

The AuNRs were prepared using the seed-mediated method [29]. CTAB (3.46 g) was added to 95 mL of warm deionised water (DW) and stirred to dissolve the powder completely. After cooling the CTAB solution to room temperature, 5 mL of the HAuCl_4_·xH_2_O (0.01 M) solution was added and mixed gently for 15 min, followed by the addition of 4.6 mL freshly prepared ice-cold NaBH_4_ (0.1 M) in NaOH (0.1 M). The growth solution was prepared by mixing the binary surfactant solution (CTAB 0.037 M and NaOL 0.23 M in 400 mL of warm DW) with 100 mL of 0.01 M HAuCl_4_·xH_2_O, followed by 2 mL of AgNO_3_ (0.1 M) and 1.8 mL HCl (12.1 M) under gentle stirring. Finally, 25 mL HQ (0.1 M) and a 100 mL seed solution were added under continuous stirring. The solution was left uninterrupted for 16−20 h at room temperature, followed by purification and characterisation.

### 2.5. Synthesis of Graphene Oxide-Coated Gold Nanorods

To fabricate the GO@AuNRs nanocomposite, PVP (16 mg) was added to GO dispersion (4 mL, 1 mg/10mL in H_2_O), followed by stirring for 30 min. Then, 2 mL of AuNRs dispersion was added to the PVP-stabilised GO solution under vigorous stirring for 24 h at room temperature. The resulting dispersion was washed and centrifuged three times to remove excess PVP and AuNRs. The final pellet was dissolved in 2 mL of deionised water.

### 2.6. Synthesis of Meso-Tetra-(4-sulfonatophenyl)porphyrin (TPPS_4_)

The meso*-*tetra-(4-sulfonatophenyl)porphyrin (TPPS_4_) was synthesised using our previously reported method [30], by firstly preparing TPPH_2_ as an intermediate porphyrin (UV-Vis spectrum λ: Soret band (410 nm) and Q-bands (512 nm, 549 nm, 590 nm, and 648 nm); PL emission λ: 650 nm and 730 nm with excited wavelength 512 nm) and it was then sulphonated using H_2_SO_4_ to produce TPPS_4_.

### 2.7. Conjugation of TPPS_4_ to GO@AuNRs

The formation of GO@AuNRs nanocomposite-porphyrin (GO@AuNRs-TPPS_4_) conjugate was carried out using our previous method [12] with modifications. Briefly, 0.2 mL of TPPS_4_ (0.1 mg/mL) was added to 2 mL of GO@AuNRs and stirred for 1 h at room temperature. The solution was characterised then freeze-dried and stored for further analysis.

### 2.8. Freeze-Drying of AuNRs, GO@AuNRs, and GO@AuNRs-TPPS_4_

The AuNRs, GO@AuNRs, and GO@AuNRs-TPPS_4_ were frozen by adding 15 mL of each sample in 50 mL centrifuge tubes. They were frozen for 24 h at −80 °C in an ultra-low temperature deep freezer (Meling −86 °C Ultra-low Temperature Freezer DW-HL50, Zhongke Meiling Cryogenics Co., Ltd., Anhui, China) before the freeze-drying process, which lasted for 48 h. The freeze dryer (MRC, FDL-10-50-Bench top freeze dryer, Wirsam Scientific and Precision Equipment (Pty) Ltd., Johannesburg, South Africa) was set at −50 °C with a pressure of 0.1 mbar. The freeze-dried samples were then characterised. Triplicates were performed for all samples in this work.

### 2.9. Photothermal Evaluation

The temperature changes were measured using an RS-1384 PRO thermocouple. The photothermal efficiency of water, GO@AuNRs, and GO@AuNRs-TPPS_4_ was measured by placing 3 mL of water, GO@AuNRs solution, and GO@AuNRs-TPPS_4_ solution separately in a UV-Vis quartz cuvette followed by irradiation under a continuous 808 laser with a power density of 1.27 W·cm^−2^. The temperature changes were measured using the RS-1384 PRO thermocouple. The photothermal conversion efficiency (PCE) was evaluated by irradiating 3 mL GO@AuNRs or GO@AuNRs-TPPS_4_ with 808 nm power density of 1.27 W·cm^−2^ until a constant temperature was reached, and then the laser system was switched off. The temperature of the dispersions was recorded every 30 s using a thermocouple. The PCE was calculated following Li et al.’s calculation with modifications [31].

### 2.10. Fluorescence Quantum Yield Measurements

TPPS_4_ and GO@AuNRs-TPPS_4_ were dispersed in distilled water to have an absorbance close to 0.1 at 560 nm, while methylene blue (MB) was dispersed in water to have an absorbance close to 0.1 at 560 nm. The samples were placed in a 3 mL pre-cleaned quartz cuvette with 1 cm optical path. The entire spectrum was scanned against the background spectrum of water. The emission spectra and quantum yield (QY) were obtained at ambient conditions using a Shimadzu RF-6000 (Japan) spectrophotometer. The QY was calculated using the following Equation (1):

(1) $${QY}_{s}=\left(\frac{\left(\frac{A_{s}}{A_{MB}}\right)\times \left(\frac{{Abs}_{MB}}{{Abs}_{s}}\right)}{\left(\frac{n_{MB}^{2}}{n_{s}^{2}}\right)}\right)\times {QY}_{MB},$$

where QY_S_ and QY_MB_ are the quantum yields of the sample and reference, A_S_ and A_MB_ are the area under the high-intensity peaks of the sample and reference, Abs_S_ and Abs_MB_ are the absorbances at the excitation wavelength of the sample and reference, and n_S_ and n_MB_ are the refractive indexes of the sample and reference, respectively. The reference standard used was the MB solution in water with QY of 0.52.

### 2.11. Singlet Oxygen Quantum Yield Evaluation

Singlet oxygen quantum yield (SOQY) was determined following Tsolekile et al. [30]. Briefly, the standard of 1,3-diphenyl benzofuran (DPBF) solution (32 mM in DMSO), MB solution (20 mM in DMSO), TPPS_4_ solution (10 mM in water), and GO solution (0.1 mg/mL in water) were prepared. Each sample was separately mixed in the ratio of 1:1 with DPBF and the mixtures were irradiated using photoluminescence spectrophotometry laser light at 535 nm for 6 min with a 1.5 min time interval. The DPBF intensity decrease was monitored at 471 nm peak position. The SOQY was calculated against an aqueous solution of methylene blue (20 mM) as a standard (SOQY = 0.52) using Equation (2):

(2) $$SOQY=\left(\frac{\left(\frac{I_{s}}{I_{MB}}\right)\left(\frac{M_{s}}{M_{MB}}\right)}{\left(\frac{{Abs}_{s}}{{Abs}_{MB}}\right)}\right){SOQY}_{MB},$$

where SOQY and SOQY_MB_ are the SOQY of the samples (TPPS_4_, conjugate) and the reference (MB, 0.52), Mc and M_MB_ are the slopes of the samples and reference, Abs_s_ and Abs_MB_ are the absorbance of the samples and the reference at irradiation wavelength, and I_S_ and I_MB_ are the refractive indexes of the sample and reference, respectively.

## 3. Results

### 3.1. Characterisation of Gold Nanorods, Graphene Oxide, and Graphene Oxide-Anchored Gold Nanorods Conjugated to TPPS_4_

**Figure 1:**
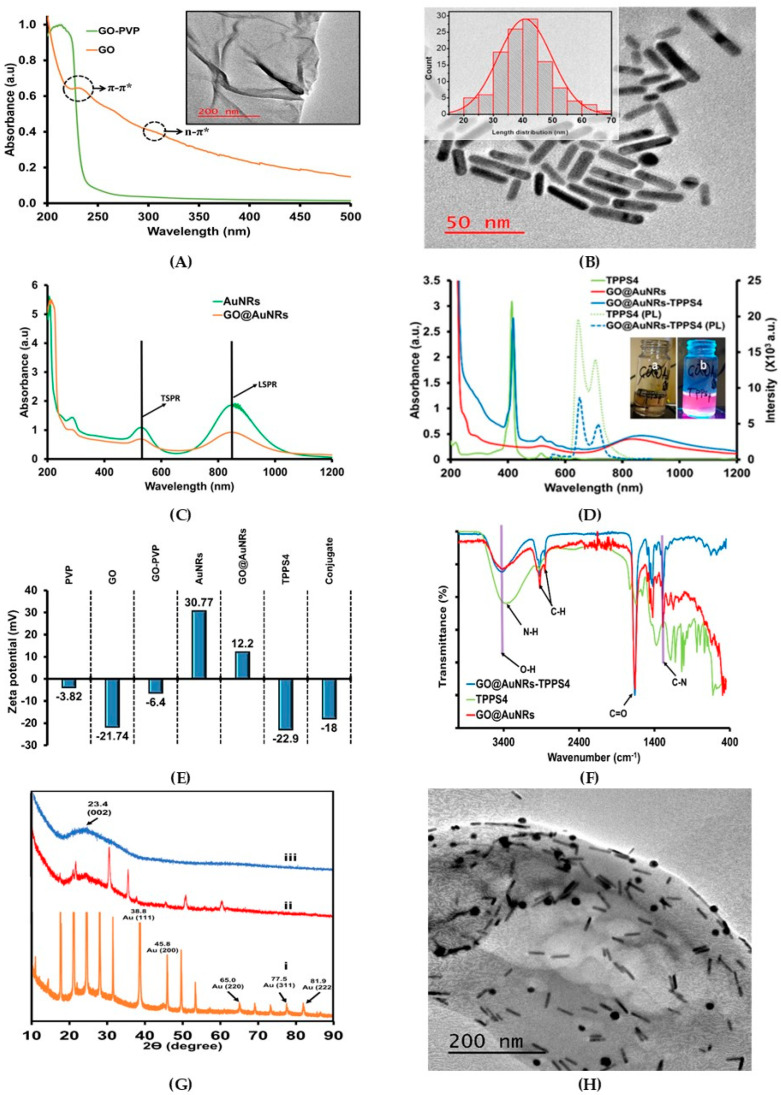
(**A**) UV-Vis spectra of GO and GO-PVP (inset: TEM image of GO-PVP, scale: 200 nm). (**B**) Bare BS-HQ-AuNRs TEM image (scale: 50 nm; inset: size distribution histogram). (**C**) UV-Vis-NIR spectra of AuNRs and GO@AuNRs. (**D**) UV-Vis-NIR and PL spectra of TPPS_4_ and GO@AuNRs-TPPS_4_ (excited at 415 nm; inset: photographic images of GO@AuNRs-TPPS_4_ under normal light (a) and UV-light irradiation (b)). (**E**) Zeta potential of GO, PVP, GO-PVP, AuNRs, GO@AuNRs, TPPS_4_, and GO@AuNRs-TPPS_4_. (**F**) FTIR spectra of TPPS_4_, GO@AuNRs, and GO@AuNRs-TPPS_4_. (**G**) XRD pattern of GO@AuNRs-TPPS_4_ (i), GO@AuNRs (ii), and AuNRs (iii). (**H**) TEM image of GO@AuNRs-TPPS_4_.

GO was synthesised using the Hummers’ method, and the aqueous dispersibility was improved by modifying it with PVP [12,32,33]. The UV spectra of the as-synthesised GO (Figure 1A) showed small shoulder peaks corresponding to π-π* carbon–carbon and n-π* oxygen–carbon transitions. When GO was modified with PVP, the π-π* and n-π* of GO disappeared, indicating the interaction of PVP with GO. The FTIR analyses of GO, PVP, and GO-PVP were performed to confirm the modification. The inset TEM image of GO-PVP in Figure 1A shows a sheet with slight folding. The AuNRs with the size range of 40.70 ± 9.1 nm × 7.31 ± 1.6 nm (Figure 1B) and absorption at 820 nm were synthesised using the seed-mediated method previously reported [29]. The AuNRs were anchored on the modified GO via electrostatic interaction. The UV-Vis-NIR of the GO-anchored AuNRs did not show changes in the transverse surface plasmon resonance (TSPR) or LSPR peak wavelength position. However, the absorbance decreased (Figure 1C). The GO-PVP peak is also seen in the GO@AuNRs UV-Vis-NIR spectrum, confirming the bend of the AuNRs and GO-PVP. The zeta potential of the AuNRs decreased from 30 mV to 12 mV after the formation of GO@AuNRs.

The GO@AuNRs were conjugated to TPPS_4_ via electrostatic stacking. The UV-Vis-NIR spectrum of GO@AuNRs-TPPS_4_ shows the TSPR of AuNRs merged with the Q-bands of porphyrin. In addition, a slightly red-shifted longitudinal surface plasmon resonance (LSPR) of AuNRs as well as the Soret peak of TPPS_4_ can be observed (Figure 1D). Unlike our previous paper on 5,10,15,20-tetrakis (3-methyl pyridyl) porphyrin conjugated to GO@AuNRs [12], the Soret band was slightly red shifted and according to the literature, this red shift is due to the H-aggregation due to the electrostatic interaction of TPPS_4_ with the AuNRs and GO [34,35]. The PL results were similar to graphene–porphyrin conjugates reported [36,37,38,39,40,41,42,43]. However, there was a drastic emission reduction because of the red shift in the UV-Vis spectra. This could be attributed to the weak interaction of TPPS_4_ with GO@AuNRs surface via the coordination interaction between gold or graphene and pyrrole nitrogen atoms [15]. The GO@AuNRs-TPPS_4_ sample emits pink-red under UV light (Figure 1D). In addition, the zeta potential of the GO@AuNRs-TPPS_4_ was negatively charged (−18.0 mV) due to the negatively charged TPPS_4_ (−22.9 mV) anchored on the surface of AuNRs (Figure 1E). The FTIR spectrum of GO@AuNRs-TPPS_4_ (Figure 1F) exhibits most of the GO@AuNRs bands, such as alkyl C-H bands, OH broadband, C=O, and C-O stretching vibration at ~2850 and ~2918 cm^−1^, ~3424 cm^−1^, ~1667 cm^−1^, and ~1240 cm^−1^, respectively. The OH band in GO@AuNRs-TPPS_4_ was more stretched. However, the TPPS_4_ vibration peaks of SO_3_^2–^ at 1124 cm^−1^ and 1198 cm^−1^ in GO@AuNRs-TPPS_4_ are not visible because they are covered by GO. The XRD of the bare-AuNRs shows diffraction peaks at 17.9°, 21.4°, 24.8°, 28.2°, and 31.6° corresponding to the (002), (102), (210), (301), and (310) for CTAB [44], whereas, the diffraction peaks at 38.8°, 45.8°, 65.0°, 77.5°, and 81.9° are attributed to (111), (200), (220), (311), and (222) of Au (JCPDS file: 04-0784) (Figure 1G(i)). The AuNRs diffraction peaks in the GO@AuNRs are almost covered—only a few peaks can be seen (Figure 1G(ii)). However, after conjugation they completely disappeared. These results suggest that the AuNRs are completely sandwiched between GO and porphyrin; thus, the XRD spectrum shows only the strong broad diffraction peak at 23.4°, which corresponds to (002), the characteristic peak of amorphous carbon (Figure 1G(iii)).

**Figure 2:**
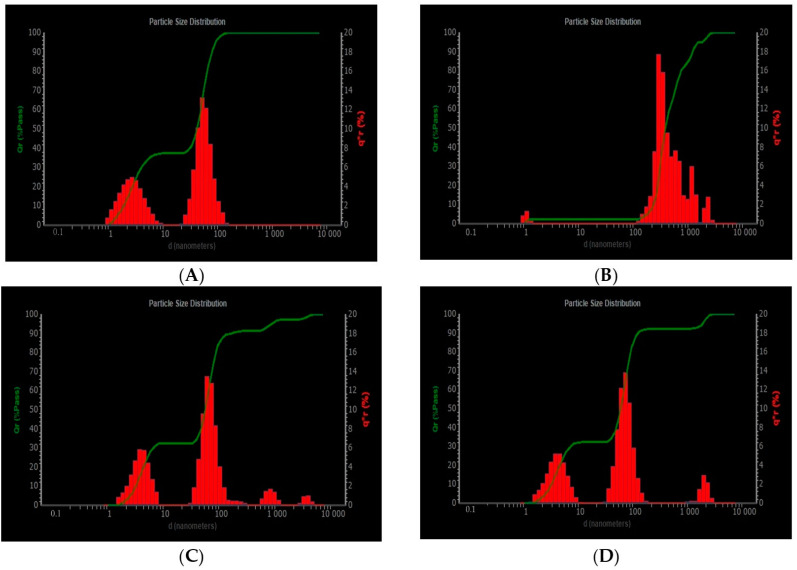
DLS hydrodynamic size of (**A**) AuNRs, (**B**) GO, (**C**) GO@AuNRs, and (**D**) GO@AuNRs-TPPS_4_.

The TEM of GO@AuNRs-TPPS_4_ in Figure 1G shows a GO sheet decorated with AuNRs. The DLS of AuNRs, GO, GO@AuNRs, and GO@AuNRs-TPPS_4_ were evaluated. Figure 2 shows similar AuNRs hydrodynamic size (Figure 2A) in both the GO@AuNRs (Figure 2C) and GO@AuNRs-TPPS_4_ (Figure 2D) in the range of 49.9 ± 9.2 nm. The difference between these and AuNRs is that in both the GO@AuNRs and GO@AuNRs-TPPS_4_, another peak is seen at ~1000 nm, which is attributed to GO (Figure 2B). This might be the reason behind the similar TEM micrographs observed for both GO@AuNRs-TPPS_4_ and GO@AuNRs.

### 3.2. Lyophilisation of Nanocomposite Conjugated to Porphyrin

**Figure 3:**
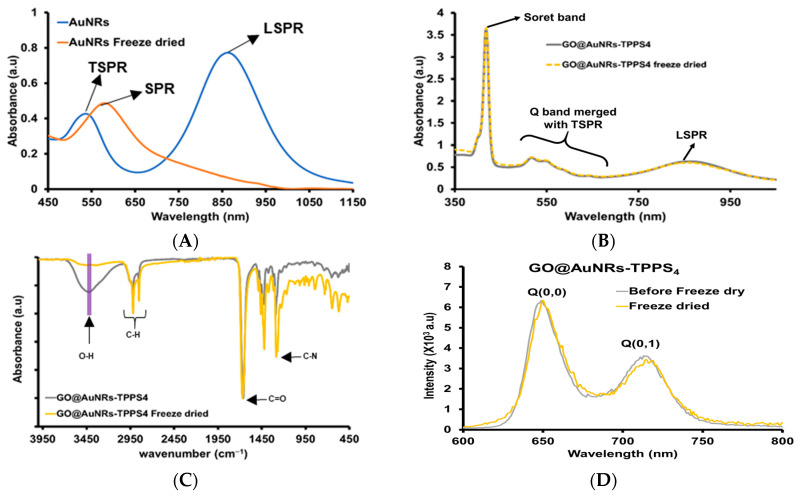
(**A**) UV-Vis-NIR spectra of AuNRs before and after freeze-drying. (**B**) UV-Vis-NIR spectra of GO@AuNRs-TPPS_4_ before and after freeze-drying. (**C**) FTIR spectra of GO@AuNRs-TPPS_4_. (**D**) PL of GO@AuNRs-TPPS_4_ before and after freeze-drying (excited at 560 nm).

The colloidal stability of GO@AuNRs-TPPS_4_ upon free drying was tested and compared to the AuNRs and GO@AuNRs. The AuNRs synthesised were freeze-dried to maintain their colloidal stability. We noticed during the course of the study that AuNRs were not stable in water with time. However, the AuNRs lost their shape after freeze-drying, as shown by the UV-Vis-NIR spectrum in Figure 3A. After freeze-drying, the AuNRs LSPR disappeared as the only visible peak was the TSPR, which was broad at 585 nm; similar results were observed by Khlebtsov et al. [22]. The AuNRs were then coated with GO at a 1:2 ratio; however, the solid mass obtained was not dispersible in deionised water, making it difficult to capture its UV-Vis as the solids were just settling down in the deionised water. According to Ham et al. [24], the oxygen-containing functional groups are removed during the freeze-drying process. Studies have shown that the oxygen-containing functional groups on the graphene are the reason for the easy dispersibility of GO in water [24]. In contrast to both the AuNRs and mGO@AuNRs, the mGO@AuNRs-TPPS_4_ was soluble in water as TPPS_4_ after freeze-drying. TPPS_4_ has been reported to improve the solubility of nanomaterials such as quantum dots [30]. The UV-Vis-NIR spectrum of freeze-dried GO@AuNRs-TPPS_4_ shows all the peaks of both the TPPS_4_ and AuNRs, the Soret, Q band, and LSPR with no shift compared to the GO@AuNRs-TPPS_4_ before freeze-drying (Figure 3B). These results confirm that there was no aggregation of the AuNRs and TPPS_4_. The FTIR of the freeze-dried GO@AuNRs-TPPS_4_ in Figure 3C displayed all the GO@AuNRs-TPPS_4_ peaks seen before freeze-drying. However, the OH peak was less pronounced in the freeze-dried GO@AuNRs-TPPS_4_. The PL of the freeze-dried GO@AuNRs-TPPS_4_ was performed, which also shows similar peaks as the unfreeze-dried GO@AuNRs-TPPS_4_ with the Q(0,0) and Q(0,1) (Figure 3D)_._ This result shows that GO@AuNRs-TPPS_4_ can be lyophilised without losing its characteristics and was due to the TPPS_4_. Similar studies have shown that porphyrins can improve solubility of graphene materials [25].

### 3.3. Lifetime, Quantum Yield, and Singlet Oxygen Generation Profiling

**Figure 4:**
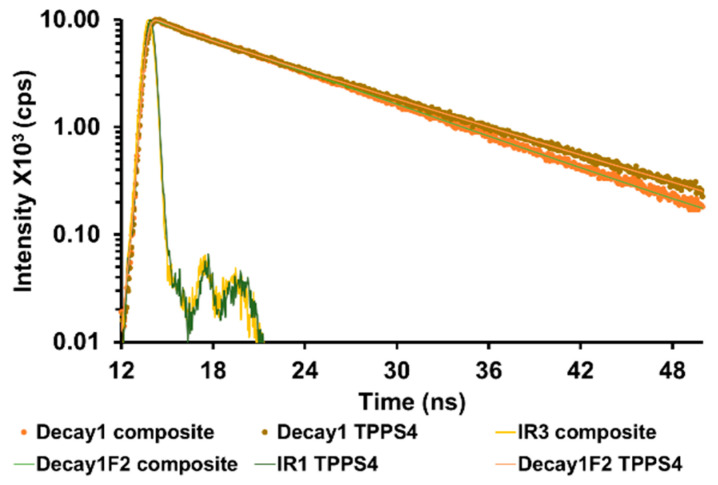
PL lifetime of TPPS_4_ and GO@AuNRs-TPPS_4_.

The relative fluorescence quantum yield of TPPS_4_ and GO@AuNRs-TPPS_4_ were analysed using PL and UV-Vis-NIR spectroscopy. The UV-Vis-NIR spectroscopy was used to obtain the absorbance lower than 0.1 a.u or close to 0.1 a.u for all the samples at 560 nm, which is the excitation wavelength. All the samples were excited at 560 nm, and methylene blue (MB) was used as the reference. The area under the curve of MB, TPPS_4_, and GO@AuNRs-TPPS_4_ was obtained from the PL emission spectrum when all the samples were excited at 560 nm. The relative fluorescence quantum yield of TPPS_4_ and GO@AuNRs-TPPS_4_ were 1.14 and 0.32, respectively. The relative fluorescence quantum yield was reduced by 72% for TPPS_4_ after the composite formation. The 72% reduction in relative fluorescence quantum yield observed in TPPS_4_ might be due to the strong interaction of TPPS_4_ with AuNRs, which caused the TPPS_4_ to H-aggregate. The lifetimes of the PL of TPPS_4_ and GO@AuNRs-TPPS_4_ were calculated using a time-correlated single-photon counting (TCSPC) technique with a pulsed LED at 450 nm. The lifetime of the PL intensity decays substantially as a bi-exponential function with the characteristic decay time average (τ_avg_) of 6.11 ns and 6.75 ns (Figure 4, Table 1), respectively. The results show an increase in the decay time of GO@AuNRs-TPPS_4_ compared to TPPS_4_. This can be attributed to the electron absorption of AuNRs, which increases the electron transfer in both states and can be assigned to efficient back electron transfer and recovery of the ground state conjugate [14,43,45]. These results verify the efficient emission quenching of TPPS_4_ by the GO@AuNRs in the GO@AuNRs-TPPS_4_, as observed in the fluorescence intensity quenching in the PL spectra (Figure 1D).

**Figure 5:**
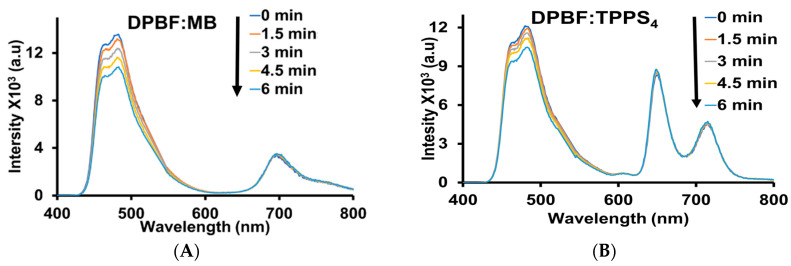

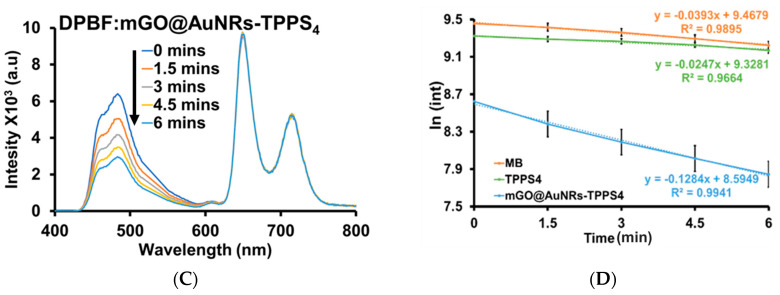
PL emission spectra of (**A**) DPBF: MB, (**B**) DPBF: TPPS_4_, and (**C**) DPBF: GO@AuNRs-TPPS_4_ excited at 560 nm for 6 min at the intervals of 1.5 min. (**D**) Integrated emission intensity time graph of DPBF at 471 nm after mixing with MB, TPPS_4_, and GO@AuNRs-TPPS_4_ and irradiated at 560 nm for different time intervals of 6 min.

The photochemical method was used to detect the singlet oxygen emission quantum yield (SOQY) of TPPS_4_ and GO@AuNRs-TPPS_4_ using DPBF as a singlet oxygen scavenger and MB as the reference. The singlet oxygen generation kinetics of the mixed samples with DPBF were acquired by irradiation at 535 nm at an interval of 1.5 min for 6 min. The PL spectrum changes of the DPBF peak at 471 nm were monitored (Figure 5A–C). The singlet oxygen generation reaction rate constants were found to be 0.013 and 0.13 min^−1^ for TPPS_4_ and GO@AuNRs-TPPS_4_, respectively, as calculated from first-order integrated rate reaction Equation (3):

(3) $${\ln\left[int\right]}_{t}={ln\left[int\right]}_{0}-kt$$

where [int]_t_ is the intensity of DPBF (471 nm) at a particular irradiation time (t), while [int]_0_ is the initial intensity at the beginning of the reaction, and k is the reaction rate constant, which is the slope of the graph.

The slopes of the graphs in Figure 5D and, together with Equation (2), were used to calculate the SOQY of TPPS_4_ and GO@AuNRs-TPPS_4_. The SOQY of GO@AuNRs-TPPS_4_ was more dramatically higher than the bare TPPS_4_, respectively. The SOQY of TPPS_4_ (0.276) almost doubled after the formation of GO@AuNRs-TPPS_4_ (0.5056).

### 3.4. Photothermal Profiling Characterisation

**Figure 6:**
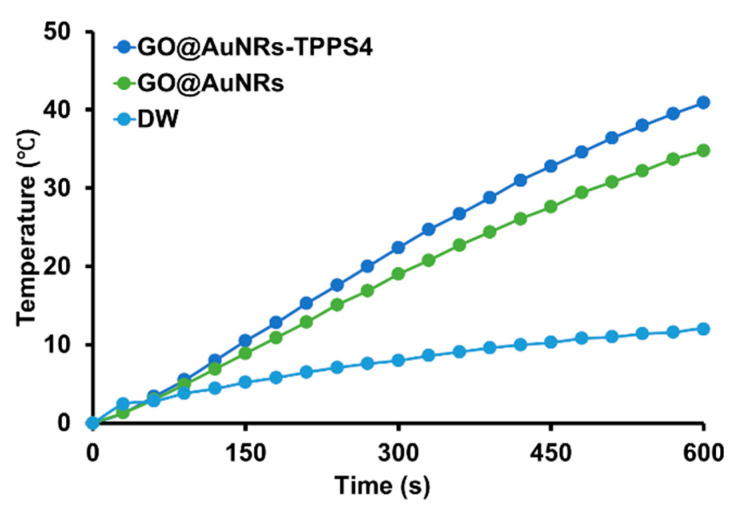
The photothermal profiling of water, GO@AuNRs, and GO@AuNRs-TPPS_4_ irradiated with 808 lasers (a power density of 1.27 W.cm^−2^).

Photothermal evaluation of water, GO@AuNRs, and GO@AuNRs-TPPS_4_ was performed using an 808 nm laser with a power density of 1.27 W.cm^−2^ for 6 min (Figure 6). The results show an increase in temperature change when irradiated with time. The water temperature increased to only 9.6 °C, while the GO@AuNRs and GO@AuNRs-TPPS_4_ increased to 38.5 and 41.0 °C, respectively.

## 4. Conclusions

In summary, the GO@AuNRs-TPPS_4_ was successfully synthesised and could tolerate freeze-drying stress without adding cryoprotectant during the freeze-drying process. The conjugate exhibited photothermal and photodynamic properties upon irradiation. The GO was synthesised using Hummers’ method and modified with PVP before being decorated with 40.70 ± 9.1 nm × 7.31 ± 1.6 nm AuNRs absorbing at 820 nm, which was synthesised via the seed-mediated method. The optical and structural characterisation methods were used to confirm the formation of GO@AuNRs. The GO@AuNRs was then conjugated to TPPS_4_. The conjugation was confirmed by optical and structural characterisation method, revealing that the AuNRs were sandwiched between the GO and TPPS_4_. The AuNRs, GO@AuNRs, and conjugate were freeze-dried. Among the freeze-dried materials, the conjugate was easily dispersible in water and kept its optical properties compared to the original sample. The freeze-dried conjugate showed an increase in the decay time compared to TPPS_4_, attributed to the electron absorption of AuNRs’ increasing electron transfer in both states. The SOQY of TPPS_4_ (0.276) increased by 55% after the formation of GO@AuNRs-TPPS_4_ (0.5056). The photothermal profiling showed that GO@AuNRs-TPPS_4_ produced more heat than the bare GO@AuNRs, which is high enough for it to be used as a photothermal agent. The results showed that GO@AuNRs-TPPS_4_ is an excellent opto-phototherapeutic agent with good dispersion after freeze-drying.

## Figures and Tables

**Table 1:** The time-resolved fluorescence decay of TPPS_4_ and GO@AuNRs-TPPS_4_.

Sample	PLQY (%)	τ_1_ (ns)	τ_2_ (ns)	χ^2^	τ_avg_ (ns)
TPPS_4_	1.14	2.36 (4.86%)	9.85 (95.14%)	1.28	6.11
GO@AuNRs-TPPS_4_	0.31	4.86 (1.89%)	8.63 (98.11%)	1.30	6.75

## Data Availability

The data presented in this study are available on request from the corresponding author.
